# Transgender Attitudes and Beliefs Scale-Greek (TABS-Gr) version: translation and initial evaluation of psychometric properties among medical students

**DOI:** 10.1186/s12909-023-04666-7

**Published:** 2023-09-27

**Authors:** Polychronis Voultsos, Angeliki Papana, Stella Alexandri, Christina-Erato Zymvragou

**Affiliations:** 1 Laboratory of Forensic Medicine & Toxicology (Division: Medical Law and Ethics), School of Medicine, Faculty of Health Sciences, Aristotle University, University Campus, Thessaloniki, GR 54124 Greece; 2 School of Mathematics, Aristotle University, University Campus, Thessaloniki, GR 54124 Greece

**Keywords:** Transgender health, Discrimination, Health inequities, Transgender Attitudes and Beliefs Scale (TABS), Medical education, Medical curriculum

## Abstract

**Background:**

Transgender people face significantly greater discrimination and health disparities in health care settings than cisgender people. The role of education in eliminating this phenomenon has been increasingly recognized by many medical schools. However, transgender health content is sparse or lacking in the medical curricula of many countries.

**Method:**

This study was designed to validate the Greek version of the Transgender Attitudes and Beliefs Scale (TABS-Gr). The study adopted a cross-sectional, comparative-descriptive research design. Participants (*N* = 203) were contacted through online recruitment and invited to complete an anonymous web-based survey. The data were collected between December 2022 and February 2023.

**Results:**

The overall reliability of the TABS-Gr questionnaire was high (Cronbach’s α = 0.961, p. from Hotelling’s T-squared test < 0.000). High Cronbach’s alpha values were estimated for the three subscales, with α = 0.958 for Interpersonal Comfort, α = 0.906 for Gender Beliefs, and α = 0.952 for Human Values. Hotelling’s T-squared test confirmed that all items on the scale had the same mean (*p* < 0.001 for all subgroups). Explanatory factor analysis (EFA) demonstrated adequate fit. Convergent and discriminant validity were validated based on the estimated correlations. The three-factor structure of the Greek TABS version was confirmed. The mean total score was 155.95 (SD = 30.63), indicating that medical students had a moderately positive attitude towards transgender people. Participants showed significantly less biased (more tolerant, positive) attitudes towards transgender people on the Interpersonal Comfort (IC) and Human Value (HV) subscales than on the Sex/Gender Beliefs (SGB) subscale. A demographic comparison was conducted and demonstrated a correlation between scores and sociodemographics, except for place of origin. A statistically significant increase in the total mean score was estimated for women compared to men.

**Conclusion:**

The overall psychometric findings provide some evidence to support the validity of the Greek version of the TABS. However, we call for further validation research in Greek medical schools. Since our claims for validity are based in part on an exploratory factor analysis, a future confirmatory factor analysis (CFA) is part of our call for further validation research. While the results of this study are mostly in line with the results of previous research, some nuances were identified. These results may inform educators, medical school curricula and education policy-makers.

**Supplementary Information:**

The online version contains supplementary material available at 10.1186/s12909-023-04666-7.

## Background

Transgender and gender-diverse individuals are people whose preferred gender identity or gender roles do not align with their sex assigned at birth, or “those whose gender identity does not conform to conventional binary gender categories known as gender nonbinary or gender nonconforming” ([1, 2], p: 377). Transgender individuals do not fit perfectly into binary gender. Importantly, gender incongruence is no longer classified as a mental health disorder in the ICD-11 (International Classification of Diseases). There may be a fluid continuum between strictly male and strictly female. It is reported that 1.4 million individuals in the United States identify as transgender (0.5–0.6% of the population) [1, 3, 4]. In Canada, according to the Institute of Medicine (2011), transgender people represent 0.3–0.5% of the adult population [5]. In the UK, approximately 1% of the population identifies as transgender [6]. Transgender people therefore represent a proportion of the population to be reckoned with. Future doctors are likely to be asked to provide services to transgender individuals during their career, regardless of their specialty [5]. Future physicians should be able to provide high-quality medical treatment to a wide range of patient populations, including visible and invisible gender and sexual minorities [7].

Transgender individuals deal with explicit and implicit bias in everyday life. It is argued that while explicit bias against transgender people has become unacceptable in medical education, implicit bias (i.e., without intention) remains widespread and difficult to address [7]. A survey of 4,441 heterosexual first-year medical students conducted by Burke et al. found that nearly half (45.79%) of the respondents exhibited explicit bias, with most (81.51%) expressing at least some implicit bias against LGBT individuals [8]. The role of “implicit bias” in contributing to health care disparities among transgender individuals has been highlighted by many authors [9].

In this regard, a growing body of literature has been published on transgender health over the last 15 years that suggests that transgender people are medically underserved and experience significantly greater health inequities and challenges in accessing health care services than cisgender people [1–3, 5, 6, 10–19]. This phenomenon is even more disquieting in developing countries [13]. Even in countries such as Canada where there have been major legal advances that aim to provide better treatment for transgender people, they continue to be medically underserved [5]. In Greece, there have been legal advances in recent years towards recognizing transgender peoples’ rights. Political openness to LGBT+ people has been increasing in recent years in Greece, as reflected by greater awareness of LGBT rights (e.g., pride parades are allowed) and legal advances (e.g., LGBT individuals can openly serve in the military, and Greece passed a legal gender recognition law in 2017). In 2017, individuals who identified as transgender were given the right to achieve legal recognition of their gender identity under Law 4491/2017 irrespective of whether they had undergone surgical alteration of their genitals.

Transgender patients require health care that is tailored to their needs. Importantly, transgender people, especially seniors, face unique and population-specific health care challenges [2, 4–7, 14, 16, 17, 20–24]. Moreover, transgender people face health care challenges related to medical gender transition. Gender-affirming medical procedures may be related to a range of side effects [25, 26]. Medical transition (transitioning and endocrine or surgical gender-affirming medical procedures) is commonly accepted as an effective treatment for gender dysphoria and is associated with transgender individuals’ mental health [14]. Medical students are future clinicians and, as such, should be trained to recognize transgender people’s unique health care needs [2], leading to the elimination of health care disparities among the members of the transgender population.

### Discrimination against transgender people in health care settings

Individuals who identify as transgender or gender-diverse experience gender-based discrimination (mostly implicitly rather than explicitly), real or perceived stigma and potentially a lack of respect in health care settings at an individual or institutional level [5, 14, 27], which may make them unwilling to seek health care services [7, 14, 19, 28, 29]. Voultsos et al. conducted a qualitative study in Greece and found that trans men who go to midwifery units or birth centres to give birth are commonly bullied by health professionals [30]. According to previous studies, approximately 30% of transgender people are unwilling to seek necessary health care or delay health care due to fear of discrimination, since approximately 70% of transgender people have experienced discrimination [1, 4, 31]. Many transgender individuals are afraid of being treated in an undignified manner by health providers [16]. Furthermore, they are afraid of experiencing “direct harm, lack of appropriate care, and a hostile environment” [32] as well as “hurtful or insulting language, being denied care, and being belittled or ridiculed” [29]. Transgender individuals may experience “anti-LGBT jokes, rumours, and/or bullying” by health care providers [7]. A large survey of transgender people conducted in the US by the National Center for Transgender Equality and the National Gay and Lesbian Task Force reported that participants faced refusal of care, harassment and violence in medical settings and a lack of provider knowledge [31]. The United Nations’ Resolution A/RES/70/1 (*Transforming our world: the 2030 agenda for sustainable development*) is working towards a world without inequities [33]. Movement to put an end to health inequities among transgender people began decades ago and has been more common in Western countries than in Asian countries, largely due to religious and cultural reasons [34]. A study recently conducted in Taiwan by Lu et al. is among the first research in Asia to use a qualitative approach to explore medical students’ attitudes towards providing health care for LGBT patients [34]. The US Department of Health and Human Services’ Healthy People 2020 programme recognized these disparities and strived to put an end to them [35].

A lack of health care providers who are knowledgeable about transgender medicine is reportedly an important barrier that prevents transgender people from accessing high-quality health care services [13, 14, 36–38]. According to a survey conducted in the US, approximately 50% of transgender people have been treated by a health provider who lacked transgender-specific knowledge [4]. At the same time, health care providers may avoid asking questions about patients’ gender identity for fear of causing offence or losing trust [39].

Health care providers should be well trained to efficiently deliver gender-diverse health care [2, 5, 40]. Poor transgender health training and a lack of cultural competence among health providers are factors that cause health disparities among transgender people [6, 22, 38]. Striving to eliminate health disparities and discrimination against transgender people in health care settings requires the integration of training on gender competence in undergraduate medical education [2, 41]. Medical students may unconsciously integrate gender-related implicit biases and stereotypes as part of their medical education. Teachers serve as role models, and their conscious or unconscious biases against transgender people may play an important role in the so-called hidden curriculum and thus in the development of medical students’ negative attitudes towards transgender people [7, 34, 42]. Lu et al. concluded that students are aware of this phenomenon [34]. Cheng et al. conducted an analysis of the hidden curriculum and suggested strategies for integrating gender sensitivity into the medical curriculum [42]. Nama et al. found that medical students are often exposed to anti-trans biases “early on in their training” and described the climate at their school as “noninclusive” [7].

### Implications for medical curriculum development including transgender health content

Medical students should be systematically and explicitly provided with curricular education (through formal, informal and hidden curricula in preclinical and clinical training) on transgender cultural competency to improve their beliefs, attitudes, knowledge and willingness to care for transgender people [1, 3, 12, 15, 16, 22, 23, 34, 36, 40, 43–47].

Over the past decade, the role of education in eliminating health disparities among transgender individuals has been increasingly recognized by many medical schools, which have made efforts to include LGBT-specific content in their curricula [2, 22, 43, 48]. In the UK, the General Medical Council (GMC) suggests that medical students should understand the social determinants of health [6]. Belonging to the transgender community is a determinant of health. However, many studies have identified a gap in education on transgender health in the undergraduate curricula of medical schools. Many medical schools fail to integrate gender-diverse content in their curricular frameworks or spend only a few hours on such topics. Transgender health content is sparse or lacking (or poor) and varies considerably across the medical curricula in many countries [2, 4, 6, 10, 13, 29, 38, 49, 50].

The WHO strongly suggests the inclusion of transgender health content in the curricular frameworks of medical schools [51], as do the UK’s Medical Students Conference [24], the American Dental Education Association’s Diversity and Inclusion Advisory Committee [15], and the Association of American Medical Colleges and Centers for Disease Control and Prevention [1, 4, 13, 19, 20, 52, 53] as well as professional organizations such as the American Psychological Association (APA), American Academy of Paediatrics (AAP), American College of Obstetrics and Gynaecology (ACOG), American Urological Association (AUA), and Association of American Medical Colleges (AAMC) [1, 12]. The AAMC suggested specific transgender curricular content for medical students to promote a transgender inclusive culture and professional competence [3, 4, 15]. Furthermore, many studies suggest increased education on gender-diverse issues in medical education [2, 5, 11, 34, 43].

Most importantly, teaching LGBT health content is a task that should not be taken lightly. It requires answers to questions such as what and who should teach and how much time should be spent on this teaching [50]. In addition, there may be inadequate research on how medical students perceive LGBT patients [54]. A lack of knowledge or a lack of suitable instructors are among the reasons why medical schools do not include gender-diverse content in their curricula [2, 4, 21, 34]. Many studies suggest that longitudinal interventions based on clinical skills are more effective [2, 3, 12, 13, 15, 16, 34, 38, 55, 56]. Longitudinal transgender-specific medical education must involve a range of teaching modalities, including exposure to transgender individuals, and must be tailored to students’ needs, knowledge and perspectives [19, 40, 52, 53]. Therefore, quality assessment and result validity are required [19]. To this end, assessing transgender-related attitudes and beliefs among teachers and students might be helpful to develop effective educational interventions for transgender health [57]. In particular, the assumption is true that student-led educational interventions are particularly effective [3, 58]. Moreover, including transgender individuals in training activities, such as clinical exposure to provide care for patients who identify as transgender individuals or using interactive panels consisting of transgender community members, can further improve the effectiveness of transgender-specific medical education [2, 3, 13, 15, 16, 24, 32, 40, 59].

Unsurprisingly, studies emphasize the inclusion of transgender individuals in training activities [32, 50]. The transgender health curriculum at Boston University involves didactic sessions and clinical exposure to patients who identify as transgender people [13]. A study conducted by Levy et al. demonstrated “the effectiveness of the small-group, case-based discussion approach involving members of the LGBT community as facilitators to enhance the cultural competency of the medical students” [16]. As mentioned above, Berenson et al. developed an educational programme in which “small-group facilitators included LGBT and allied students” [40]. Importantly, Stroumsa argued that medical students’ transphobic attitudes are negatively correlated with the inclusion of transgender individuals in training activities irrespective of the number of curriculum hours spent on the topic of transgender medicine [32]. In the UK, final-year medical students believe that “the transgender community is best able to educate medical professionals” and that role-play and communication skills should be involved in such a curriculum [24].

A large number of studies have provided evidence that educational interventions, namely, incorporating transgender curricular content in mandatory (core) medical school curricula, can effectively improve medical students’ knowledge, skills, and attitudes towards transgender people as well as their comfort with transgender patients, cultural competence, confidence, and willingness to treat transgender patients, ultimately reducing health disparities against transgender patients [2–6, 11, 12, 14–23, 29, 34, 38, 42–44, 50, 52, 56, 58–60]. However, while studies have demonstrated significant improvement in students’ knowledge, skills and attitudes towards transgender patients, these findings should be interpreted with caution for a number of reasons [2].

Finally and most importantly, it should be noted that while transgender-specific medical education continues to be an area for improvement in many countries (e.g., in the US), data on this topic are scant [40]. For a number of reasons, there is no consensus regarding the structure and strategies of the most effective training programmes (e.g., duration, content and methodology) [2, 7, 21, 24, 61]. The formats of the few existing programmes vary considerably [2].

The information discussed above illustrates the need for a greater focus on good practices and an incorporation of the results into the curricula. This topic is related to social justice with respect to health. Exploring medical students’ attitudes towards transgender individuals is one of the first steps in that direction. The Transgender Attitudes and Beliefs Scale (TABS) was developed and validated in 2017 in English by Kanamori et al. and evaluates respondents’ attitudes towards transgender people [62, 63]. The TABS was intended to improve scales that researchers had previously attempted to develop to evaluate attitudes towards transgender people. The original TABS was designed to assess attitudes towards transgender people in the general population and was tested in the US general population. As the TABS was designed to assess attitudes towards transgender individuals in general (not transgender patients or health care consumers only), it can also be used to assess attitudes towards transgender individuals among medical students (as members of the general population). Although it is not yet extensively used, to the best of our knowledge, it is the only scale that evaluates in a nuanced way people’s attitudes towards (only) transgender people. In this regard, it should be noted that Kanamori et al. (2017) state, “TABS represents an addition to the literature in its ability to capture a more nuanced conceptualization of transgender attitude not found in previous scales” ([62], p: 1503).

The original validation study reported an optimal alpha coefficient of internal consistency (α = 0.98) and evidence of convergent and discriminant validity [62]. Researchers subsequently attempted to validate it in other contexts. The TABS-Spanish version has already been validated and published [64] and was used in a study with Spanish psychology students [65]. Recently, it was used in a survey conducted by Campbell et al. at a publicly supported Caribbean university [57]. Furthermore, the reliability and validity of the scale has been assessed in the general population of Mexico [66].

The TABS is a 29-item scale. Items are rated on a 7-point Likert scale ranging from 1 = strongly disagree to 7 = strongly agree, with higher scores indicating more positive attitudes towards transgender persons. As people’s attitudes towards transgender people represent a complex and multifactorial social phenomenon, the TABS is a three-factor model that includes three subscales: comfort with transgender individuals (“interpersonal comfort”, IC, 14 items), beliefs related to gender identity (“sex/gender beliefs”, SGB, 10 items), and a factor related to transgender people’s inherent human value (“human value”, HV, 5 items). Items 3, 4, 8–11, 13 and 14 from the 1^st^ factor (interpersonal comfort) and Items 1, 3–5, 8, and 9 from the 2^nd^ factor (sex/gender beliefs) are reverse scored. Reverse-scored items are used to create balanced scales where some questions are phrased in the opposite direction to prevent response biases. To analyse them in SPSS, new variables for reverse-scored values are created by subtracting the original values from the maximum possible value plus one. After this reversal, the interpretation of all items is consistent; that is, higher scores suggest a more positive attitude of medical students towards transgender people.

To the best of our knowledge, no published studies have quantitatively measured medical students’ attitudes towards transgender individuals in Greece. Fradelos et al. investigated the psychometric properties of the Nursing Students’ Knowledge of and Attitudes towards Lesbian, Gay, Bisexual, and Transgender (LGBT) Health Concerns (NKALH) survey [67]. Papadaki et al. investigated attitudes towards lesbians and gay men among students of helping professions (including medical and nursing students), using the Attitudes toward Lesbians and Gay Men (ATLG) Scale developed by G.M. Herek [68]. To fill this knowledge gap, this study was designed to validate the Transgender Attitudes and Beliefs Scale (TABS)-Greek version and to examine medical students’ attitudes towards transgender people. The results of this study are expected to give future studies the opportunity to use the validated Greek version of the TABS in the context of medical education, thus contributing to the improvement of medical education in terms of reducing gender-based health disparities.

## Research questions

The following research questions are addressed in this research:

- Is the Greek version of the Transgender Attitudes and Beliefs Scale (TABS-Gr) valid and reliable for medical students?
- What are the attitudes towards transgender people among medical students in early clinical exposure at the Aristotle University of Thessaloniki (Greece)?
- How are sociodemographic factors (including gender, sexual orientation, place of origin, religiosity/spirituality, interest in issues of medical ethics/bioethics, interest in receiving training on caring for transgender people, and comfort with transgender patients during medical education) correlated with attitudes towards transgender people among medical students in early clinical exposure at the Aristotle University of Thessaloniki (Greece)?

## Methods

### Study design

This study adopted a cross-sectional, comparative-descriptive research design. The survey was conducted with medical students of the medical school of Aristotle University of Thessaloniki (Greece). In Greece, as in most European countries, the first 3 years of medical studies are called “preclinical” and focus on basic sciences. The second 3 years are called “clinical”, as students receive training in clinical specialties. The authors of this study decided to explore students in their first “clinical” year for two reasons. Fourth-year students are in early clinical exposure. The fourth year of medical studies represents a critical stage in medical education where students are transitioning from classroom learning to clinical practice. Studying this specific cohort can yield insights into their clinical skills, ethical decision-making, preparedness for practice, and the impact of their education. It also offers a unique opportunity to evaluate the effectiveness of educational interventions and curriculum components. In this regard, it should be noted that, according to many prior studies, the levels of empathy among medical students in early clinical exposure decline noticeably. Relevant literature is cited in the study by Voultsos et al. [69]. Moreover, all participants in this study had just completed a course on medical law and ethics at the time of the survey, which is the only compulsory course on bioethics in their curriculum. Having attended the course in medical law and ethics was important for sampling because the course places considerable emphasis on human rights, ethical/moral values and social justice, including health equity and the principle of nondiscrimination. It is argued that moral disengagement predisposes people to adopt negative attitudes towards LGBT individuals [70]. Furthermore, it should be noted that the curriculum of the Medical School of the Aristotle University of Thessaloniki does not include an LGBT health curriculum. To the best of our knowledge, this is also the case for other Greek medical schools. In the various lessons that are taught in our medical school, few references are made regarding LGBT health issues, which cannot be considered to constitute education or training on these issues.

As to the validation approach, we had a sense of validity framework. To frame our work, however, we were based on the validation approach used by previous studies which have conducted validations of the TABS [62–64]. These studies have used widely accepted steps to collect scientifically sound validity evidence.

### Sampling procedure

The study population consisted of fourth-year students from Aristotle University of Thessaloniki (Greece) School of Medicine (*n* = 295). Finally, a total of 203 medical students participated in the study. There were many missing replies.

All students were invited to participate in the survey. Fourth-year medical students of a central and large Greek medical school (of Aristotle University of Thessaloniki) were contacted through online recruitment and invited to complete an anonymous web-based survey. Specifically, a questionnaire was distributed via Google Forms to students enrolled in the School of Medicine of the Aristotle University of Thessaloniki, the second largest medical school in Greece. The undergraduate curriculum of the School of Medicine of the Aristotle University of Thessaloniki did not address the topic of LGBT health issues at the time of the interviews. The data were collected between December 2022 and February 2023. To avoid bias in the study and to be in accordance with the relevant ethical standards, the recruitment of participants was conducted on a purely voluntary basis. Moreover, the confidentiality of the participants was strictly maintained.

### Research instrument

A questionnaire was administered to 295 fourth-year medical students. The participants filled out the questionnaire, which included two sections: sociodemographics and the TABS-Gr. The first section was on the respondents’ sociodemographic characteristics, including gender, sexual orientation, place of origin, religiosity/spirituality, interest in issues of medical ethics/bioethics, interest in receiving training on caring for transgender people, and comfort with transgender patients during medical education. The second section was the TABS-Gr (described above).

### Instrument translation

The authors translated the Transgender Attitudes and Beliefs Scale (TABS; [62]) into Greek using the back-translation methodology to ensure the conceptual equivalence of the scale in Greek and English and to assess the translated scale’s content validity.

There was need for consensus among the researchers on how to best translate and achieve a high-quality translation of the TABS in Greek. The forward translation of the original scale into Greek was performed independently by two experts, a native Greek speaker who had English language skills and experience translating medical questionnaires and a psychologist for whom Greek was the first language and who lived and worked in the US for many years. The psychologist culturally adapted the translated scale so that it would be more consistent with Greek language expressions. Then, the Greek version was back-translated to English independently by a professional translator. Finally, all members of the research group reviewed the final version and compared the final backward-translated version with the original English version to ensure the conceptual equivalence of the final Greek version and the original English version. The researchers discussed the resulting translations with translators (cognitive debriefing). PV, SA and C-EZ had extensive experience in bioethics, psychology and empirical research. Minor modifications were made to the Greek version of the scale after the original scale was compared with the back-translated version to make it sensitive to cultural nuances and ensure understanding for Greek speakers while achieving equivalency in meaning. The Greek version of the TABS is presented in Supplementary file 1.

### Data analysis

Data preparation and preliminary analyses were conducted using the statistical program SPSS 25.0 (Statistical Package for the Social Sciences) of IBM. A 7-point scale from “strongly disagree” to “strongly agree” was used (1: Strongly disagree, 2: Disagree, 3: Somewhat disagree, 4: Neither agree nor disagree, 5: Somewhat agree, 6: Agree, and 7: Strongly agree). To generate a useful numerical evaluation, we input the results (data) from the 7-points Likert scale using SPSS predictive analytics software. In SPSS’ “variable view”, we created different variables for each item on the Likert scale. Then, in “data view”, we entered each respondent’s answers, entering “1” for “strongly disagree”, “2” for “disagree” and so on up the scale. The ratings were converted to numbers.

Descriptive statistics were used to summarize the demographic characteristics of the participants, such as frequency distributions and percentages. Regarding the TABS-Gr items, the subscales and total scores, means and standard deviations are displayed. The total scores were extracted by adding the total from the 29 items of the TABS and ranged from 29 to 203. A higher total score on the TABS reflects more positive attitudes towards transgender people. Paired-samples t tests were used to check the statistically significant differences in total means among the three subscales. Significant differences between the subscales and the TABS-Gr from the subgroups on the demographic characteristics were established using independent-sample t tests and one-way ANOVA (analysis of variance) tests. Furthermore, we estimated Pearson’s correlation coefficients to examine the TABS-Gr subscale correlations and item correlations. The internal consistency of the subscales and of the TABS-Gr questionnaire was analysed with Cronbach’s α and Hotelling’s T-squared test, while Pearson correlation was utilized for the examination of the validity of the questionnaire. Independent-sample t tests, One-way ANOVA (Analysis of Variance) and Independent-Samples Kruskal-Wallis tests were used to examine the total score mean differences of the subgroups based on demographics. Finally, explanatory factor analysis was performed to examine the conceptual validity of the Greek version of the TABS-Gr questionnaire. The adequacy of the sample was validated with the Keiser-Meyer-Olkin (KMO) coefficient, and Bartlett’s test of sphericity was utilized to examine the applicability of factor analysis. Varimax rotation was considered for the analysis, and 3 factors were used to conduct the analysis.

## Results

Explanatory factor analysis (EFA) showed adequate fit. Convergent and discriminant validity were validated based on the estimated correlations. Therefore, the three-factor structure of the Greek TABS version was confirmed. Overall, the results suggest that the TABS-Gr is an appropriate instrument to utilize with the medical student population.

### Sociodemographics

The sample consisted of 42.4% males and 56.2% females. Approximately 88.7% of the sample were not LGBT persons. Most of the medical students were between 18 and 25 years old (89.2% of the sample). Religious participants (respondents who stated belief in God or another supreme being) constituted 66.5% of the sample. The majority of participants were interested in medical ethics/bioethics issues (84.2% of the sample). Furthermore, the majority of participants wanted to receive training on caring for transgender people (as a part of their medical curriculum) to be able to provide high-quality treatment for transgender people as physicians (70% of the sample), although 58.6% of the participants felt that they did not have sufficient knowledge and skills to approach transgender patients during their medical studies. Finally, 63.1% of the students replied that they would not feel uncomfortable if they had to approach a transgender patient. Tables 1, 2, 3, 4, 5, 6, 7, 8 and 9 show the sociodemographic information in detail.

**Table 1:** Gender

Gender	Female	Male	Something else	No answer
	56.2%	42.4%	1%	0.5%

**Table 2:** Sexual orientation

Sexual orientation	Not LGBT persons	LGBT persons	Do not know/no answer
	88.7%	7.9%	3.4%

**Table 3:** Age

Age	18–25 years	26–30 years	> 30 years
	89.2%	9.4%	1.5%

**Table 4:** Degree of urbanization of the area where participants grew up

Grew up	Small town (< 100.000 residents)	Large town (> 100,000 residents	Large city (e.g., Athens or Thessaloniki)
	32%	21.2%	32%

**Table 5:** Religiosity

Religious	Yes	No	Do not know/no answer
	66.5%	17.7%	15.8%

**Table 6:** Interest in the field of medical ethics/bioethics

Interested in medical ethics/bioethics	Yes (quite interested)	No (interested)	Slightly interested	Not at all interested
	60.1%	24.1%	14.8%	1%

**Table 7:** Willingness to receive training on transgender medicine

Willingness to receive training on transgender medicine	Yes	No	Do not know/no answer
	70%	16.7%	13.3%

**Table 8:** Self-assessment of knowledge and skills to care for transgender individuals

Students consider themselves to have sufficient knowledge and skills to care for transgender individuals	Yes	No	Do not know/no answer
	26.6%	58.6%	14.8%

**Table 9:** Comfort with transgender patients

Students feel comfortable in approaching transgender patients	Yes	No	Do not know/no answer
	63.1%	24.6%	12.3%

### TABS-Gr items

Table 10 displays the descriptive statistics for each item of the TABS-Gr. The sample showed accepting attitudes towards transgender people, with the mean values of items concerning Interpersonal Comfort varying from 4.33 to 6.07. The mean values of items concerning Sex/Gender Belief were slightly above the midpoint (4.11–5.21). The highest mean scores were obtained for the items that concerned Human Value (6.50–6.63).

**Table 10:** Descriptive statistics for items of TABS-Gr scales. Items with reversed scores are noted with superscript ^R^

Interpersonal Comfort			Sex/Gender Belief			Human Value		
Item	Mean	SD	Item	Mean	SD	Item	Mean	SD
Q1.1	5.43	1.56	Q2.1^R^	5.21	1.70	Q3.1	6.51	0.84
Q1.2	5.21	1.71	Q2.2	4.30	1.80	Q3.2	6.63	0.76
Q1.3^R^	5.63	1.48	Q2.3^R^	4.68	1.80	Q3.3	6.58	0.83
Q1.4^R^	5.47	1.72	Q2.4^R^	5.06	1.57	Q3.4	6.50	0.84
Q1.5	5.87	1.35	Q2.5^R^	4.11	1.93	Q3.5	6.61	0.80
Q1.6	6.07	1.20	Q2.6	5.09	1.67			
Q1.7	5.38	1.68	Q2.7	4.53	1.76			
Q1.8^R^	4.33	1.79	Q2.8^R^	4.26	1.80			
Q1.9^R^	5.77	1.42	Q2.9^R^	4.48	1.57			
Q1.10^R^	4.43	1.89	Q2.10	4.75	1.79			
Q1.11^R^	5.72	1.45						
Q1.12	5.91	1.40						
Q1.13^R^	5.53	1.53						
Q1.14^R^	5.71	1.49						

Significant Pearson’s correlation coefficients were estimated between the TABS-Gr items, with r varying from 0.20 to 0.88 (*p* < 0.05)

### Explanatory factor analysis

Explanatory factor analysis confirmed the conceptual validity of the Greek version of the TABS questionnaire. The adequacy of the sample was validated with a high Keiser-Meyer-Olkin (KMO) coefficient (0.947). Bartlett’s test of sphericity supported the implementation of factor analysis (*p* < 0.05).

Three factors were requested in the analysis since the items were designed to index three constructs: Interpersonal Comfort, Sex/Gender Beliefs, and Human Value. Utilizing the Varimax rotation, the analysis showed that the 3 factors explained 67.17% of the total data variability. The 1st factor, however, explained over half of the total variability (50.27%).

Based on the analysis, the items were assigned to the three factors as shown below.

- ✓ **Factor 1** (Interpersonal Comfort): Q1.1–Q1.14, Q2.6
- ✓ **Factor 2** (Human Value): Q2.1–Q2.5, Q2.7–Q2.10
- ✓ **Factor 3** (Sex/Gender Beliefs): Q3.1–Q3.5

Communalities are estimates of the common variance shared between each observed variable and the factors. Items with very low communalities could be omitted from the analysis as long as they contribute meaningfully to the interpretation of factors. The estimated communalities of the items are presented in Table 11. The communalities varied from 0.917 (E3.2) to 0.258 (E2.10).

**Table 11:** Estimated communalities from factor analysis

Item	Communalities	Item	Communalities
E3.2	.917	E2.7	.723
E3.5	.890	E1.14	.692
E3.3	.860	E1.13	.684
E3.1	.830	E1.11	.681
E1.7	.802	E1.10R	.672
E1.5	.800	E2.6	.629
E2.5	.796	E2.2	.580
E1.9R	.791	E2.1	.514
E1.6	.770	E1.8R	.511
E1.1	.751	E1.12	.499
E1.2	.751	E2.4	.453
E2.3	.748	E1.4R	.366
E1.3R	.740	E2.9	.306
E3.4	.734	E2.10	.258
E2.8	.729		

All loadings were positive. The strongest loadings were specified for the 3^rd^ factor (Human Value), varying from 0.70 to 0.93. Most items of the 1st factor (Interpersonal Comfort) also had high loadings, varying from 0.54 to 0.83, while the 2^nd^ factor (Sex/Gender Beliefs) had the lowest loading (from 0.35 to 0.86). Items E1.2, E1.7, E1.8, E1.10, E1.13, E1.14, E2.1, E2.3, E2.6, E2.8, and E2.10 had positive loadings for both the 1^st^ and 2^nd^ factors, while E1.11 had positive loadings for the 1^st^ and 3^rd^ factors (see Table 12).

**Table 12:** Loadings from factor analysis (rotated factor matrix) with loadings of each factor in descending order

Item	1	2	3
Factor 1: Interpersonal Comfort			
Q1.6	**0.836**		
Q1.5	**0.834**		
Q1.1	**0.800**		
Q1.3^R^	**0.795**		
Q1.9^R^	**0.794**		
Q1.2	**0.777**	0.320	
Q1.13^R^	**0.751**	0.338	
Q1.7	**0.739**	0.459	
Q1.14^R^	**0.727**	0.313	
Q1.11^R^	**0.704**		0.359
Q1.10^R^	**0.656**	0.462	
Q1.12	**0.624**		
Q2.6	**0.604**	0.456	
Q1.8^R^	**0.546**		
Q1.4^R^	**0.543**		
Factor 2: Human Value			
Q2.5^R^		**0.864**	
Q2.7		**0.806**	
Q2.3^R^	0.365	**0.756**	
Q2.8^R^	0.387	**0.752**	
Q2.2		**0.709**	
Q2.4^R^		**0.610**	
Q2.1^R^	0.417	**0.562**	
Q2.9^R^		**0.499**	
Q2.10	0.314	**0.353**	
Factor 3: Sex/Gender Beliefs			
Q3.2		**0.934**	
Q3.5		**0.911**	
Q3.3		**0.894**	
Q3.1		**0.871**	
Q3.4		**0.790**	

Explanatory factor analysis demonstrated acceptable overall fit for the three-factor scale.

### TABS-Gr subscales

The internal consistency of the subscales was confirmed by reliability analysis. High Cronbach’s alpha values were estimated for the three subscales, with α = 0.958 for Interpersonal Comfort, α = 0.906 for Gender Beliefs, and α = 0.952 for Human Value. Hotelling’s T-squared test confirmed that all items on the scale had the same mean (*p* < 0.001 for all subgroups).

Because people’s attitude towards transgender individuals is a complex and multifactorial social phenomenon and Interpersonal Comfort, Sex/Gender Belief and Human Value are three factors that play a substantial role in medical students’ training on transgender-specific health issues and transgender cultural competency, it is important to report the differences between the TABS-Gr subscales. The subscale scores were computed as the sum of all the items of each subscale and varied from 14 to 98 for Interpersonal Comfort, from 10 to 70 for Gender Beliefs, and from 5 to 35 for Human Beliefs. The corresponding mean total scores were 76.53 (SD = 17.56), 46.47 (SD = 12.84) and 32.83 (SD = 3.73) for the three subscales respectively, suggesting increased Interpersonal Comfort of students with transgender people, an almost neutral attitude towards Gender Beliefs and high sensitivity of students regarding Human Beliefs. Additionally, paired samples t tests showed that there was a statistically significant difference in the total means among the three subscales (*p* < 0.001).

Finally, significant Pearson’s correlation coefficients were estimated between the TABS-Gr subscales (*p* < 0.05); specifically, the Interpersonal Comfort subscale was highly correlated with Sex/Gender Beliefs (*r* = 0.77) and moderately correlated with Human Values (*r* = 0.50), and Sex/Gender Beliefs was moderately correlated with Human Values (*r* = 0.42). Construct validity was demonstrated by significant correlations between the TABS-Gr subscales.

### Demographic comparison

The overall reliability of the TABS-Gr questionnaire was high (Cronbach’s α = 0.961, p. from Hotelling’s T-squared test < 0.000). The total scores for the 29 items of the TABS-Gr ranged from 29 to 203. The mean total score was 155.95 (SD = 30.63), indicating that medical students had a moderately positive attitude towards transgender people.

Independent t tests and one-way ANOVA tests were conducted and demonstrated the mean total score differences of subscales by sociodemographic variables, except for place of origin. An increased total mean score was estimated for females (165.95, SD. = 25.33) compared to males (142.81, SD = 32.06). Statistically significant mean differences were also found between religious (149.29, SD = 30.94) and nonreligious students (173.58, SD = 22.71) or students who did not know/answer about religiosity/spirituality (164.25, SD = 27.66). Significantly different score means are obtained in terms of the interest of participants in issues of medical ethics/bioethics (with higher mean scores for students more interested in bioethics), in terms of receiving training on caring for transgender people and in terms of feeling comfortable with transgender patients during medical education.

The following three Tables (Tables 13, 14, and 15) display the significant mean differences of overall scores of the TABS-Gr for sociodemographic characteristics, i.e., for interest in issues of medical ethics/bioethics, interest in receiving training on caring for transgender people, and feeling comfortable with transgender patients during medical education, respectively. We present the differences in the total TABS scores for these education-related variables with the aim of informing educators, medical school curricula and education policy-makers about developing strategies to address gender-based health care disparities. In addition, we present these differences in the hope that this finding might serve as a starting point for further research. To the best of our knowledge, literature on these differences is lacking.

**Table 13:** Mean total scores of the TABS-Gr for interest in issues of medical ethics/bioethics

Interest on medical ethics/bioethics	Mean	SD
A lot	159.06	31.84
Quite a bit	158.43	29.61
A little	143.52	29.92
Not at all	119.00	4.24

**Table 14:** Mean total scores of the TABS-Gr for interest in receiving training on caring for transgender people

Interest in receiving training on caring for transgender people	Mean	SD
Interested	166.92	23.90
Not interested	125.36	33.38
Do not know/no answer	136.00	22.68

**Table 15:** Mean total scores of the TABS-Gr for comfort with transgender patients during medical education

Comfort of participants with transgender patients	Mean	SD
Uncomfortable	162.34	29.60
Comfortable	143.88	30.74
Do not know/no answer	147.12	27.11

Furthermore, women had significantly increased mean total scores compared to men and an increased positive attitude towards transgender people (*p* < 0.001). Table 16 displays the mean total scores for each subscale for women and men.

**Table 16:** Mean total scores for each subscale of the TABS-Gr by gender

Gender	Interpersonal Comfort		Sex/Gender Belief		Human Value	
	Mean	SD	Mean	SD	Mean	SD
Female	82.15	14.05	50.25	10.99	33.46	2.94
Male	69.24	19.12	41.31	13.31	32.09	4.29

Statistically significant mean differences were found for the subscales of Interpersonal Comfort and Sex/Gender Belief depending on whether the participants were religious (Table 17).

**Table 17:** Mean total scores of each subscale of the TABS-Gr for religiosity/spirituality

Religiosity/Spirituality	Interpersonal Comfort		Sex/Gender Belief		Human Value	
	Mean	SD	Mean	SD	Mean	SD
Not religious	86.19	11.55	54.02	11.51	33.36	3.41
Religious	72.78	18.34	43.86	12.64	32.64	3.86
Do not know/no answer	81.52	14.67	49.71	11.12	33.03	3.67

Significantly different score means were obtained for the Interpersonal Comfort subscale and the Human Value subscale in terms of the participants’ interest in issues of medical ethics/bioethics (Table 18). The score means were also significantly different for the three subscales in terms of the participants’ interest in receiving special training to better treat transgender people (Table 19). Finally, significantly different mean scores were obtained for the Interpersonal Comfort subscale and the Sex/Gender Belief subscale in terms of comfort with transgender patients during medical education (Table 20).

**Table 18:** Mean total scores of each subscale of the TABS-Gr for interest in issues of medical ethics/bioethics

Interest in medical ethics/bioethics	Interpersonal Comfort		Sex/Gender Belief		Human Value	
	Mean	SD	Mean	SD	Mean	SD
A lot	78.30	17.86	47.49	13.46	33.26	4.66
Quite a bit	77.97	16.99	47.25	12.90	33.21	2.87
A little	69.10	17.90	42.86	11.41	31.43	3.63
Not at all	58.50	3.53	40.00	0.00	20.50	0.70

**Table 19:** Mean total scores of each subscale of the TABS-Gr for the participants’ interest in receiving training on caring for transgender people

Interest in receiving training on caring for transgender people	Interpersonal Comfort		Sex/Gender Belief		Human Value	
	Mean	SD	Mean	SD	Mean	SD
Not interested	60.21	19.74	34.60	12.41	30.54	5.48
Interested	82.46	13.88	50.70	10.98	33.76	2.65
Do not know/no answer	65.51	14.17	39.70	10.14	30.78	4.16

**Table 20:** Mean total scores of each subscale of the TABS by comfort with transgender patients during medical education

Participants’ comfort with transgender patients	Interpersonal Comfort		Sex/Gender Belief		Human Value	
	Mean	SD	Mean	SD	Mean	SD
Uncomfortable	68.31	19.54	48.67	12.92	32.91	3.90
Comfortable	80.77	15.93	42.81	11.85	32.76	3.49
Do not know/no answer	71.12	14.53	43.82	12.23	32.64	3.55

In addition, since a very small sample of LGBT people participated in the study, we conducted non-parametric Independent-Samples Kruskal-Wallis Test, which indicated statistically significant differences between participants that replied YES and NO regarding the variable “LGBT person” (*p* = 0.049) (See Table 21).

**Table 21:** Pairwise comparisons of the variable “LGBT person” based on the Independent-Samples Kruskal-Wallis Test

Sample 1 - Sample 2	Test Statistic	Std. Error	Std. Test Statistic	Sig	Adj. Sig.^a^
NO-Do not answer	-4.705	22.411	-.210	.834	1.000
NO-YES	-36.406	15.180	-2.398	.016	.049
Do not answer-YES	31.701	26.356	1.203	.229	.687

Each row tests the null hypothesis that the Sample 1 and Sample 2 distributions are the same

Asymptotic significances (2-sided tests) are displayed. The significance level is .050

^a^Significance values have been adjusted by the Bonferroni correction for multiple tests

## Discussion

The psychometric evaluation of the TABS-Gr demonstrated high internal consistency that provides support for the overall reliability of the scale (Cronbach’s α = 0.961) and for the three subscales (α = 0.958 for IC, α = 0.906 for S/GB and α = 0.952 for HV). Campbell et al. recently presented a psychometric evaluation of the “promising instrument” of the TABS and provided support for further use of the scale in the Caribbean. They found that “internal consistency was strong for the total TABS (α = 0.93) and more variable for the three subscales: interpersonal comfort (IC: α = 0.91), sex/gender beliefs (SGB: α = 0.89), and human value (HV: α = 0.74)”. Explanatory factor analysis demonstrated acceptable overall fit for the three-factor model [57]. Kanamori et al. found “preliminary evidence to suggest that the TABS-S is a valid and reliable scale appropriate for use with Spanish-speaking populations”. These authors found that “the internal consistency of the TABS-S scores was high for the total scale (α = 0.96) and subscales (α = 0.87–0.96)” [64]. In addition, it should be noted that in Greece, Fradelos et al. assessed the psychometric properties of the Nursing Students’ Knowledge of and Attitudes toward LGBT Health Concerns (NKALH) survey in Greece [67]. In that study the total Cronbach’s alpha value was found to be 0.783, namely, almost similar to Cronbach’s alpha value of the original version (Cronbach’s alpha = 0.77) [67].

### Medical students demonstrated positive attitudes towards transgender individuals and comfort in providing care for them

In the present study, the mean total score of the TABS-Gr was 155.95 (SD = 30.63), indicating that medical students demonstrated a moderately positive attitude towards transgender people. In this regard, 63.1% of the students replied that they would feel comfortable if they had to approach a transgender patient.

These findings are in line with previous literature. In prior studies, students with positive attitudes towards transgender individuals reported that they felt comfortable with individuals who identified as transgender. Below, we contextualize the findings within previous research. In this study, the participants reported significantly less biased (more tolerant, positive) attitudes towards transgender people on the Interpersonal Comfort (IC) and Human Value (HV) subscales than on the Sex/Gender Beliefs (SGB) subscale. Interestingly, Campbell et al. examined the attitudes and beliefs of Caribbean medical students towards transgender people using the Transgender Attitudes and Beliefs Scale (TABS) and found that students reported significantly more tolerant attitudes towards transgender people on the HV subscale than on the IC or SGB subscales [57]. Bunting et al. conducted a survey-based study and found that medical students demonstrated positive attitudes towards sexual and gender minority people [71]. In a survey conducted by Martins et al. in Pakistan (Aga Khan University), “most students demonstrated good (49.4%) or fair (45.0%) attitudes towards individuals who identified as transgender” [13]. Moreover, “The majority of students reported that they were comfortable around individuals who identify as transgender (65.5%), that they would be comfortable treating a patient who identified as transgender (87.6%), and that they believed individuals who identify as transgender should be treated with the same respect and dignity as any other person (95.2%)” ([13], p:409). Nama et al. found low bias against LGBT persons among medical students at the University of Ottawa [7]. Importantly, the respondents believed that LGBT persons are born this way at a much higher rate (79.6%) than the general population. However, while these students felt comfortable treating most LGBT persons, they felt less comfortable providing care to transgender patients [7]. Nowaskie et al. explored 940 medical students’ LGBT cultural competency at three universities across the United States and found that participants in their study reported “very high attitudinal awareness, moderate knowledge, and low clinical preparedness” ([43], p: 442(5)). In a similar vein, Arthur et al. conducted a survey of medical students in the UK and concluded that while their attitudes towards LGBT individuals were positive, they showed a lack of knowledge of LGBT patients’ specific health problems and deficits in confidence in treating these patients [6]. In Greece, Fradelos et al. found that nursing students had a positive attitude toward caring for LGBT patients [67]. Papadaki et al. found a slightly positive attitude toward lesbians and gay men among social work, psychology, medical, and nursing students in Crete, Greece, using Herek’s ATLG scale [68]. Interestingly, medical students scored lower than psychology students or social work students on positive attitudes toward lesbians and gay men [68].

In this regard, it has to be noted that feeling uncomfortable with transgender individuals does not always mean a less positive attitude towards them. While high rates of positive attitudes among medical students towards transgender people reinforce their interest in providing care for that population, inadequate preparation may cause medical students to feel low levels of comfort with transgender patients. Chan et al. found that while participants (medical students) in their study had (unanimous) interest in providing care to transgender people, they felt a low comfort level with transgender patients because of inadequate preparation [5]. Park et al. found that while the participants (medical students) in their study were interested in caring for transgender people before attending an elective transgender-specific curriculum, they initially felt a lack of comfort with transgender patients, which increased from 45 to 80% after attending the transgender-specific curriculum [3]. Muns et al. reported that while medical students at the University of Puerto Rico Medical School were willing to treat transgender patients, their knowledge and training on the topic of transgender health was limited [72]. In a similar vein, Vasudevan et al. conducted a survey of medical students at a single US academic health centre and found that while students exhibited a high degree of personal comfort in providing treatment for transgender patients, their knowledge and skills regarding the topic of transgender health were limited [73]. Greene et al. conducted a study with medical, dental and nursing students and found that while most participants (70–74%) felt comfortable treating LGBTQ patients, 71–81% of them reported interest in receiving formal education on LGBTIQ health issues [15].

Given that Voultsos et al. recently found moderately high empathy among medical students at the same medical school [69], the assumption that there may be a positive correlation between levels of empathy and positive attitudes towards transgender individuals among medical students remains to be examined. In this regard, it must be noted that researchers recently found a positive correlation between empathy and positive attitudes towards transgender individuals [8, 70, 74].

Finally, it should be noted that in Greece, citizens are less positive towards LGBTIQ persons than in many other European countries [75]. This may partly explain why the participants in this study demonstrated moderately rather than strongly positive attitudes towards transgender people.

### Medical students’ awareness of a lack of education on transgender-specific health issues and desire for further education on these issues

While the medical students who participated in this study demonstrated a moderately positive overall attitude towards transgender people, the majority (70%) of them expressed a desire to receive further education on transgender medicine (as part of their medical curriculum) to be able to provide high-quality treatment for transgender people as physicians (in the future). Furthermore, 58.6% of the respondents were aware of their lack of knowledge and skills to approach transgender patients during their medical studies. These findings are in line with the findings of previous studies.

Many studies conducted at medical schools have reported that students were aware of their lack of LGBT health education and expressed a need for further education on transgender health issues during their studies [2, 7, 17, 37, 43, 54]. For instance, Rambarran et al. conducted a survey with students at a medical school in the Caribbean (Guyana) and concluded that most students expressed a desire to receive LGBT-specific medical education and had not previously received this education [54]. Chan et al. found that in Canada, approximately 70% of medical students at the University of British Columbia did not feel that the topic of transgender-specific health was “proficiently taught” [5]. In Tollemache et al.’s study, their participants (medical students) reported that they felt dissatisfied in general with the education they received on LGBT issues at their institutions [21]. In a survey conducted in the US, 15% of students from one medical school declared that they had not received any training on LGBT health issues [45]. In the UK, final-year medical students stated that a lack of LGBT health training in medical curricula was completely unacceptable and could reinforce the marginalization of transgender people [24]. Surprisingly, Martins et al. found a “high perceived need for trans-health curricula” among medical students in Pakistan, although they demonstrated deficiencies in knowledge on transgender health issues. The majority of students “reported a high (54.6%) or moderate (42.2%) perceived need for the inclusion of trans-health in the medical curriculum” ([13], p: 405). Furthermore, Nama et al. found a consensus among students in favour of the need for further education on LGBT issues and their desire to receive such education [7]. Studies have shown that most medical students or physicians are unaware of or feel unprepared to address topics related to medical gender transitioning and hormonal therapies for transgender-identified individuals [5, 11, 15, 40]. Lu et al. found that students “expressed wide social acceptance and openness towards LGBT individuals, but were unsure of ways to communicate with LGBT patients” ([34], p: 1/16, e0270862).

In connection with the aforementioned information, it should be noted that a systematic review very recently conducted by Yu et al. highlights that health professionals’ training in LGBTQ + cultural competency can improve health professionals’ attitudes toward LGBTQ + individuals and reduce the gender-based health disparities [76].

### The association between religiosity/spirituality and attitude towards transgender individuals

In this study, medical students with higher levels of religiosity/spirituality demonstrated less positive attitudes towards transgender individuals. Voultsos et al. found a positive correlation between empathy and religiosity among medical students at the same medical school of the Aristotle University of Thessaloniki [69]. While it is argued that empathy may be positively correlated with both religiosity and positive attitudes towards transgender individuals, the literature suggests that religiosity may be an antecedent and predictor of less positive attitudes towards transgender people [77]. Religious people are likely to believe that positive attitudes towards transgender individuals violate the values of their religion [77]. Note, however, that “more empirical exploration is needed to fully understand that nuances of the religion-transprejudice relationship” ([77], p: 21). At any rate, the relationship between religiosity and negative attitudes towards LGBT people is a consistent finding, as discussed below.

Prior studies have suggested that being religious may be associated with less positive attitudes towards LGBT persons [28, 54, 71]. Bunting et al. conducted a survey with 1007 medical students at 12 US medical schools using the Attitudes Towards LGBT People Scale. Interestingly, the authors found that religiosity/spirituality was the most significant factor that affected students’ attitudes towards transgender individuals (on all scales), and high levels of religiosity/spirituality were positively correlated with less positive attitudes towards transgender people [71]. Recently, Rambarran et al. conducted a survey with students at a medical school in the Caribbean (Guyana) and concluded that religiosity and heterosexual orientation were positively correlated with negative attitudes towards transgender individuals [54]. Szél et al. conducted a survey of students at four Hungarian medical universities and found that not being religious were associated with less negative attitudes towards sexual minorities [28]. In Greece, Papadaki et al. found that religiosity is a significant factor influencing participants’ attitudes [68].

### The association between gender and attitude towards transgender individuals

In this study, a statistically significant increase in the total mean score (indicating a more positive attitude towards transgender individuals) was estimated for women compared to men. This is in line with the currently available literature. Lee et al. concluded that being female and having transgender individuals as peers or previous education on transgender issues helps to create more positive attitudes towards transgender people [14]. Moreover, other studies have concluded that being female and having close LGBTQ acquaintances or being an LGBT person are associated with more positive attitudes towards gender and sexual minorities [28, 54, 71]. Recently, Campbell et al. examined the attitudes and beliefs of Caribbean medical students towards transgender people using the Transgender Attitudes and Beliefs Scale (TABS) and did not find significant gender differences in overall attitudes towards transgender persons, with female students reporting higher IC scores [57]. In Greece, Papadaki et al. found that gender is a significant factor influencing participants’ attitudes [68]. Furthermore, Konstantinidis et al. conducted a study with undergraduate university students of Physical Education and Sport Science (Democritus University of Thrace, Greece) using the Herek’s Attitudes toward Lesbians and Gay Men (ATLG) Scale. The researchers found gender-related differences in participants’ attitudes towards gay men only [78].

As mentioned above, Voultsos et al. found moderately high empathy among medical students at the same medical school, with women demonstrating significantly higher empathy scores than men [69]. If the abovementioned assumption (supported in the literature) that empathy may serve as an antecedent of people’s positive attitudes towards transgender individuals is accurate, empathy may be a factor that moderates the correlation between female gender and positive attitudes towards transgender individuals. In this case, the gender differences in attitudes towards transgender individuals found in this study would be consistent with the predominant trend (in the literature) of gender differences in empathy. Relevant literature is cited by Voultsos et al. [69].

### The association between sexual orientation and attitude towards transgender individuals

The analysis showed statistically significant differences between participants that replied YES and NO regarding the variable “LGBT person”. The higher mean and median total score of participants replying YES (mean 171.44, SD 30.02, median 185) compared to those replying NO (mean 154.44, SD 34.34, median 157) indicates a more positive disposition towards transgender individuals This result is in line with prior studies [28, 54, 71]. However, we should keep in mind that as being transgender is not a state of sexuality but a description of gender, some cis LGB individuals may be gender essentialists and as such may not support and respect transgender individuals who may display transphobia and bigotry [79].

## Strengths and limitations

The TABS has not yet been used in many cultural contexts. Further robust studies should confirm its effectiveness. Furthermore, some items may function differently for young students than they do for older people who have more experience of interactions with other people. Some key concepts may be too difficult for young students to understand. The small sample size limited the generalizability of the conclusions of this study. Another limitation of this study is that it was conducted in a single Greek medical educational setting. The participants were from the same medical education setting. Although there are no remarkable differences between the students of various Greek medical schools, a multicenter research study is needed in the future. Furthermore, selection bias cannot be excluded. Students who participated in this study might be less biased against transgender people. A strength of this study is that it was conducted at a medical school of a central and large Greek university with high diversity among the students.

## Implications for future research and policies regarding medical education

The use of the TABS-Gr can contribute to the development of transgender health curricula and the evaluation of the educational effectiveness of these curricula. Evaluating future doctors’ attitudes towards a gender minority (the transgender population) can significantly contribute to providing the evidence required to persuade universities to prioritize curriculum changes that support transgender inclusive practices and make changes to their curriculum to ensure that future physicians are in a position to provide high-quality health care to transgender patients. The TABS-Gr can help educators develop medical curricula designed to address health care disparities and discrimination towards vulnerable members of society. The validation of the Greek version of the TABS allows the scale to facilitate the evaluation of outcomes of training programmes designed to improve medical students’ knowledge, skills and attitudes towards transgender people. Furthermore, a mixed-method approach involving a quantitative empirical study (conducted using the TABS-Gr) and a qualitative empirical study (conducted using semistructured interviews) could be utilized to further examine medical students’ perceptions of transgender people. This could lead to a more comprehensive understanding of medical students’ attitudes towards transgender individuals and the development of strategies to make these attitudes more positive. The outcomes of mixed-method studies will inform undergraduate medical curricula and help medical education keep pace with social progress. Finally, the association of sociodemographic and other variables with medical students’ attitudes towards transgender people (which emerged within this study) could inform changes in the existing medical curriculum of the School of Medicine of the Aristotle University of Thessaloniki, Greece, with the aim of putting an end to gender-based health care disparities in the future.

## Conclusions

The overall psychometric findings provide some evidence to support the validity of the Greek version of the TABS. However, we call for further validation research in Greek medical schools. Since our claims for validity are based in part on an exploratory factor analysis, a future confirmatory factor analysis (CFA) is part of our call for further validation research. Furthermore, larger samples would enable more robust validity testing. The participants in this study showed a moderately positive attitude towards transgender individuals. A demographic comparison was conducted and demonstrated correlations between scores and most sociodemographics and other education-related variables. A statistically significant increase in the total mean score was estimated for women compared to men. Future research is recommended to investigate the relationships between medical students’ self-reported attitudes towards transgender individuals and sociodemographic or other (e.g., education-related) variables. For the most part, the results of this study are in line with the results of previous research. However, we identified some nuances that might serve as a starting point for future research. The results of this study may inform educators, medical school curricula and education policy-makers.

### Supplementary Information

**Additional file 1.**

## Data Availability

The transcripts of the full interviews that were collected and qualitatively analysed in the current study are not available for reasons of confidentiality. The redacted transcripts that were used and analysed as part of the current study can be made available by the corresponding author upon reasonable request.

## Abbreviations

TABS: Transgender Attitudes and Beliefs Scale
TABS-Gr: Transgender Attitudes and Beliefs Scale-Greek version
NKALH: Nursing Students’ Knowledge of and Attitudes toward LGBT Health Concerns scale
ATLG: Attitudes toward Lesbians and Gay Men scale
IC: Interpersonal Comfort
SGB: Sex/Gender Beliefs
HV: Human Value
EFA: Explanatory Factor Analysis

## References

[1] Cherabie J, Nilsen K, Houssayni S (2018). Transgender health medical education intervention and its effects on beliefs, attitudes, comfort, and knowledge. Kans J Med.

[2] Dubin SN, Nolan IT, Streed CG, Greene RE, Radix AE, Morrison SD (2018). Transgender health care: improving medical students’ and residents’ training and awareness. Adv Med Educ Pract.

[3] Park JA, Safer JD (2018). Clinical exposure to transgender medicine improves students’ preparedness above levels seen with didactic teaching alone: a key addition to the Boston University model for teaching transgender healthcare. Transgend Health.

[4] Wright D, Campedelli A (2016). Pilot study: increasing medical student comfort in transgender gynecology. MedEdPublish.

[5] Chan B, Skocylas R, Safer JD (2016). Gaps in transgender medicine content identified among Canadian medical school curricula. Transgend Health.

[6] Arthur S, Jamieson A, Cross H, Nambiar K, Llewellyn CD (2021). Medical students’ awareness of health issues, attitudes, and confidence about caring for lesbian, gay, bisexual and transgender patients: a cross-sectional survey. BMC Med Educ.

[7] Nama N, MacPherson P, Sampson M, McMillan HJ (2017). Medical students’ perception of lesbian, gay, bisexual, and transgender (LGBT) discrimination in their learning environment and their self-reported comfort level for caring for LGBT patients: a survey study. Med Educ Online.

[8] Burke SE, Dovidio JF, Przedworski JM, Hardeman RR, Perry SP, Phelan SM (2015). Do contact and empathy mitigate bias against gay and lesbian people among heterosexual first-year medical students? A report from the medical student CHANGE study. Acad Med.

[9] McDowell MJ, Goldhammer H, Potter JE, Keuroghlian AS. Strategies to Mitigate Clinician Implicit Bias Against Sexual and Gender Minority Patients. Psychosomatics. 2020;61(6):655–61. 10.1016/j.psym.2020.04.021. 10.1016/j.psym.2020.04.021 32641233

[10] Bleasdale J, Wilson K, Aidoo-Frimpong G, Gabriel SJ, Przybyla SM. Lesbian, gay, bisexual, and transgender (LGBT) health education in healthcare professional graduate programs: a comparison of medical, nursing, and pharmacy students. J Homosex. 2022:1–14. 10.1080/00918369.2022.2111535. Epub ahead of print. PMID: 35984396. 10.1080/00918369.2022.2111535 PMC10804216 35984396

[11] Minturn MS, Martinez EI, Le T, Nokoff N, Fitch L, Little CE (2021). Early intervention for LGBTQ health: a 10-hour curriculum for preclinical health professions students. MedEdPORTAL.

[12] Nolan IT, Blasdel G, Dubin SN, Goetz LG, Greene RE, Morrison SD (2020). Current state of transgender medical education in the United States and Canada: update to a scoping review. J Med Educ Curric Dev.

[13] Martins RS, Saleh R, Kamal H, Gillani M, Merchant AAH, Munir MM (2020). The need for transgender healthcare medical education in a developing country. Adv Med Educ Pract.

[14] Lee SR, Kim MA, Choi MN, Park S, Cho J, Lee C (2021). Attitudes toward transgender people among medical students in South Korea. Sex Med.

[15] Greene MZ, France K, Kreider EF, Wolfe-Roubatis E, Chen KD, Wu A (2018). Comparing medical, dental, and nursing students’ preparedness to address lesbian, gay, bisexual, transgender, and queer health. PLoS One.

[16] Levy A, Prasad S, Griffin DP, Ortega M, O’Malley CB (2021). Attitudes and knowledge of medical students towards healthcare for lesbian, gay, bisexual, and transgender seniors: impact of a case-based discussion with facilitators from the community. Cureus.

[17] Lee SA, O’Brien OF, Turner MJ, Kennelly MM (2022). Implementing medical student teaching on gynaecological healthcare of transgender patients. Ir Med J.

[18] Click IA, Mann AK, Buda M, Rahimi-Saber A, Schultz A, Shelton KM (2020). Transgender health education for medical students. Clin Teach.

[19] Najor AJ, Kling JM, Imhof RL, Sussman JD, Nippoldt TB, Davidge-Pitts CJ (2020). Transgender health care curriculum development: a dual-site medical school campus pilot. Health Equity.

[20] Morris M, Cooper RL, Ramesh A, Tabatabai M, Arcury TA, Shinn M (2019). Training to reduce LGBTQ-related bias among medical, nursing, and dental students and providers: a systematic review. BMC Med Educ.

[21] Tollemache N, Shrewsbury D, Llewellyn C (2021). Que(e) rying undergraduate medical curricula: a cross-sectional online survey of lesbian, gay, bisexual, transgender, and queer content inclusion in UK undergraduate medical education. BMC Med Educ.

[22] Dale VH, Philomin R (2022). Educating for quality transgender health care: a survey study of medical students. Educ Health (Abingdon).

[23] Congdon AP, Tiene K, Price C, Dufresne RL (2021). Closing the gap: raising medical professionals’ transgender awareness and medical proficiency through pharmacist-led education. Ment Health Clin.

[24] James S, Colling SH (2018). Transgender health and its current omission from medical school curriculum: medical students’ perspective. Adv Med Educ Pract.

[25] Nota NM, Wiepjes CM, de Blok CJM, Gooren LJG, Kreukels BPC, den Heijer M (2019). Occurrence of acute cardiovascular events in transgender individuals receiving hormone therapy. Circulation.

[26] Tordoff DM, Wanta JW, Collin A, Stepney C, Inwards-Breland DJ, Ahrens K (2022). Mental health outcomes in transgender and nonbinary youths receiving gender-affirming care. JAMA Netw Open.

[27] Romanelli M, Lindsey MA (2020). Patterns of healthcare discrimination among transgender help-seekers. Am J Prev Med.

[28] Szél Z, Kiss D, Török Z, Gyarmathy VA (2020). Hungarian medical students’ knowledge about and attitude toward homosexual, bisexual, and transsexual individuals. J Homosex.

[29] Underman K, Giffort D, Hyderi A, Hirshfield LE (2016). Transgender health: a standardized patient case for advanced clerkship students. MedEdPORTAL.

[30] Voultsos P, Zymvragou CE, Karakasi MV, Pavlidis P (2021). A qualitative study examining transgender people’s attitudes towards having a child to whom they are genetically related and pursuing fertility treatments in Greece. BMC Public Health.

[31] Grant JM, Mottet LA, Tanis J, Harrison J, Herman JL, Keisling M. Injustice at Every Turn: A Report of the National Transgender Discrimination Survey. Washington: National Center for Transgender Equality and National Gay and Lesbian Task Force; 2011.

[32] Stroumsa D (2014). The state of transgender health care: policy, law, and medical frameworks. Am J Public Health.

[33] United Nations (UN). Resolution A/RES/70/1. Transforming our world: the 2030 agenda for sustainable development. In: Seventieth United Nations General Assembly, New York, 13–5 September 2016. New York: United Nations. 2015. https://www.un.org/en/development/desa/population/migration/generalassembly/docs/globalcompact/A_RES_70_1_E.pdf. Accessed 24 Sept 2023.

[34] Lu PY, Hsu ASC, Green A, Tsai JC (2022). Medical students’ perceptions of their preparedness to care for LGBT patients in Taiwan: is medical education keeping up with social progress?. PLoS One.

[35] US Department of Health and Human Services. Healthy people 2020: lesbian, gay, bisexual, and transgender health. 2020. https://www.healthypeople.gov/2020/topics-objectives/topic/lesbian-gay-bisexual-and-transgender-health. Accessed 10 Aug 2023.

[36] Korpaisarn S, Safer JD (2018). Gaps in transgender medical education among healthcare providers: a major barrier to care for transgender persons. Rev Endocr Metab Disord.

[37] Arora M, Walker K, Luu J, Duvivier RJ, Dune T, Wynne K. Education of the medical profession to facilitate delivery of transgender health care in an Australian health district. Aust J Prim Health. 2019. 10.1071/PY19102. Epub ahead of print. PMID: 31738874. 10.1071/PY19102 31738874

[38] Gibson AW, Gobillot TA, Wang K, Conley E, Coard W, Matsumoto K (2020). A novel curriculum for medical student training in LGBTQ healthcare: a regional pathway experience. J Med Educ Curric Dev.

[39] Rider GN, McMorris BJ, Gower AL, Coleman E, Brown C, Eisenberg ME (2019). Perspectives from nurses and physicians on training needs and comfort working with transgender and gender-diverse youth. J Pediatr Health Care.

[40] Berenson MG, Gavzy SJ, Cespedes L, Gabrani A, Davis M, Ingram K (2020). The case of Sean Smith: a three-part interactive module on transgender health for second-year medical students. MedEdPORTAL.

[41] Papadaki V, Ntiken A (2023). “As a trans person you don’t live. You merely try to survive and apologize every day for who you are” - discrimination experiences among trans individuals in Greece. J Homosex.

[42] Cheng LF, Yang HC (2015). Learning about gender on campus: an analysis of the hidden curriculum for medical students. Med Educ.

[43] Nowaskie DZ, Patel AU (2020). How much is needed? Patient exposure and curricular education on medical students’ LGBT cultural competency. BMC Med Educ.

[44] Jeon SY, Yoon HB, Park JE, Lee SY, Yoon JW (2022). A qualitative study on the internal response of medical students during the transgender healthcare education: a focus on professional identity. Korean J Med Educ.

[45] Parameshwaran V, Cockbain BC, Hillyard M, Price JR (2017). Is the lack of specific lesbian, gay, bisexual, transgender and queer/questioning (LGBTQ) health care education in medical school a cause for concern? Evidence from a survey of knowledge and practice among UK medical students. J Homosex.

[46] Hana T, Butler K, Young LT, Zamora G, Lam JSH (2021). Transgender health in medical education. Bull World Health Organ.

[47] de Vries E, Kathard H, Müller A (2020). Debate: why should gender-affirming health care be included in health science curricula?. BMC Med Educ.

[48] Sawning S, Steinbock S, Croley R, Combs R, Shaw A, Ganzel T (2017). A first step in addressing medical education curriculum gaps in lesbian-, gay-, bisexual-, and transgender-related content: the University of Louisville Lesbian, Gay, Bisexual, and Transgender Health Certificate Program. Educ Health (Abingdon).

[49] Obedin-Maliver J, Goldsmith ES, Stewart L, White W, Tran E, Brenman S (2011). Lesbian, gay, bisexual, and transgender-related content in undergraduate medical education. JAMA.

[50] Ellaway RH, Thompson NL, Temple-Oberle C, Pacaud D, Frecker H, Jablonski TJ (2022). An undergraduate medical curriculum framework for providing care to transgender and gender diverse patients: a modified Delphi study. Perspect Med Educ.

[51] Growing recognition of transgender health. Bull World Health Organ. 2016; 94(11):790-1.10.2471/BLT.16.021116. PMID: 27821880; PMCID: PMC5096349. 10.2471/BLT.16.021116 PMC5096349 27821880

[52] Sanchez K, Abrams MP, Khallouq BB, Topping D (2022). Classroom instruction: medical students’ attitudes toward LGBTQI^+^ patients. J Homosex.

[53] Christensen JA, Hunt T, Elsesser SA, Jerpbak C (2019). Medical student perspectives on LGBTQ health. PRiMER.

[54] Rambarran N, Simpson J, White Y (2022). Medical students’ attitudes towards, and knowledge of LGBT persons in Guyana. J Homosex.

[55] Braun HM, Garcia-Grossman IR, Quiñones-Rivera A, Deutsch MB (2017). Outcome and impact evaluation of a transgender health course for health profession students. LGBT Health.

[56] Thompson H, Coleman JA, Iyengar RM, Phillips S, Kent PM, Sheth N (2020). Evaluation of a gender-affirming healthcare curriculum for second-year medical students. Postgrad Med J.

[57] Campbell MH, Gromer J, Emmanuel MK, Harvey A (2022). Attitudes toward transgender people among future Caribbean doctors. Arch Sex Behav.

[58] Grosz AM, Gutierrez D, Lui AA, Chang JJ, Cole-Kelly K, Ng H (2017). A student-led introduction to lesbian, gay, bisexual, and transgender health for first-year medical students. Fam Med.

[59] Katz-Wise SL, Jarvie EJ, Potter J, Keuroghlian AS, Gums JN, Kosciesza AJ, et al. Integrating LGBTQIA + community member perspectives into medical education. Teach Learn Med. 2022:1–15. 10.1080/10401334.2022.2092112. Epub ahead of print. PMID: 35766109. 10.1080/10401334.2022.2092112 35766109

[60] Song AY, Poythress EL, Bocchini CE, Kass JS (2018). Reorienting orientation: introducing the social determinants of health to first-year medical students. MedEdPORTAL.

[61] Sekoni AO, Gale NK, Manga-Atangana B, Bhadhuri A, Jolly K (2017). The effects of educational curricula and training on LGBT-specific health issues for healthcare students and professionals: a mixed-method systematic review. J Int AIDS Soc.

[62] Kanamori Y, Cornelius-White JHD, Pegors TK, Daniel T, Hulgus J (2017). Development and validation of the transgender attitudes and beliefs scale. Arch Sex Behav.

[63] Kanamori Y, Fossett S, Schimmel-Bristow A, Stenersen MR, Bullard MB, Cornelius-White JHD (2021). Transgender Attitudes and Beliefs Scale (TABS): validation with a sample of self-identified Christians. Ment Health Relig Cult.

[64] Kanamori Y, Jiménez-Etxebarria E, Cornelius-White JHD, Ozamiz-Etxebarria N, Wynne KN, Gorrotxategi MP (2023). Transgender Attitudes and Beliefs Scale-Spanish (TABS-S) version: translation and initial evaluation of psychometric properties. J Homosex.

[65] López-Sáez MÁ, García-Dauder D, Montero I (2020). Correlate attitudes toward LGBT and sexism in Spanish psychology students. Front Psychol.

[66] Fresan A, Domínguez-Martínez T, Castilla F, Muñoz C (2022). Confirmatory factor analysis of the Transgender Knowledge, Attitudes, and Beliefs (T-KAB) scale for the Mexican population. Arch Sex Behav.

[67] Fradelos EC, Montegrico J, Cornelius J, Bakalis V, Malliarou M, Papathanasiou IV (2022). Translation and validation of nursing students’ knowledge and attitudes of lesbian, gay, bisexual, transgender health concerns survey in the Greek language. Healthcare (Basel).

[68] Papadaki V, Plotnikof K, Gioumidou M, Zisimou V, Papadaki E (2015). A comparison of attitudes toward lesbians and gay men among students of helping professions in Crete, Greece: the cases of social work, psychology, medicine, and nursing. J Homosex.

[69] Voultsos P, Chatzinikolaou F, Papana A, Deliligka A (2022). Reliability of Greek version of the Toronto empathy questionnaire in medical students and associations with sociodemographic and lifestyle factors. BMC Psychol.

[70] Inmaculada FA, Isabel CG (2021). Discrimination and violence due to diversity of sexual orientation and gender identity: explanatory variables. Int J Environ Res Public Health.

[71] Bunting SR, Chirica MG, Ritchie TD, Garber SS, Batteson TJ (2021). A national study of medical students’ attitudes toward sexual and gender minority populations: evaluating the effects of demographics and training. LGBT Health.

[72] Muns SM, Ortiz-Ramos KJ, García-Rivera EJ, González L, Romaguera J (2022). Knowledge and attitudes about transgender healthcare: exploring the perspectives of Hispanic medical students. P R Health Sci J.

[73] Vasudevan A, García AD, Hart BG, Kindratt TB, Pagels P, Orcutt V (2022). Health professions students’ knowledge, skills, and attitudes toward transgender healthcare. J Community Health.

[74] Esteban Mora J, Morales Rodríguez FM, Martínez Ramón JP (2022). Attitudes toward transsexuality, empathy, and bullying in young population. Int J Environ Res Public Health.

[75] European Commission. Eurobarometer on the social acceptance of LGBTIQ people in the EU-2019. 2019. https://commission.europa.eu/strategy-and-policy/policies/justice-and-fundamental-rights/combatting-discrimination/lesbian-gay-bi-trans-and-intersex-equality/eurobarometer-social-acceptance-lgbtiq-people-eu-2019_en. Accessed 10 Aug 2023.

[76] Yu H, Flores DD, Bonett S, Bauermeister JA (2023). LGBTQ + cultural competency training for health professionals: a systematic review. BMC Med Educ.

[77] Campbell M, Hinton JDX, Anderson JR (2019). A systematic review of the relationship between religion and attitudes toward transgender and gender-variant people. Int J Transgend.

[78] Konstantinidis C, Bebetsos E, Filippou F, Zetou E (2021). Gender-related attitudes toward homosexuality in Greece. Chang Soc Pers.

[79] Trans Hub. Why are trans people part of LGBT? ACON 2021. https://www.transhub.org.au/. Accessed 14 Aug 2023.

