# Supplementary material for: Transgender Attitudes and Beliefs Scale-Greek (TABS-Gr) version: translation and initial evaluation of psychometric properties among medical students
Source: BMC Med Educ. 2023 Sep 27;23:704. doi: 10.1186/s12909-023-04666-7 (PMC10523621; doi:10.1186/s12909-023-04666-7)
Supplement: Supplementary file 1 — Additional file 1. [file 12909_2023_4666_MOESM1_ESM.docx]

# **Κλίμακα με τον τίτλο: Πεποιθήσεις και στάσεις απέναντι σε διεμφυλικά άτομα [Transgender Attitudes and Beliefs Scale (TABS)].**

**ΠΑΡΑΓΟΝΤΑΣ 1 (Διαπροσωπική άνεση)**

Ε1.1 Θα αισθανόμουν άνετα να κάνω το τραπέζι στο σπίτι μου σε ένα διεμφυλικό άτομο.

Ε1.2 Θα αισθανόμουν άνετα σε μια παρέα διεμφυλικών ατόμων.

Ε1.3 Δεν θα αισθανόμουν άνετα εάν ο προϊστάμενός μου ήταν διεμφυλικό άτομο.

Ε1.4 Δεν θα αισθανόμουν άνετα στη δουλειά μου να εργάζομαι κοντά σε ένα διεμφυλικό άτομο.

Ε1.5 Ακόμη και αν γνώριζα ότι κάποιος είναι διεμφυλικό άτομο, θα εξακολουθούσα να μην έχω πρόβλημα να δημιουργήσω φιλική σχέση μαζί του.

Ε1.6 Θα αισθανόμουν άνετα εάν ο γείτονας της διπλανής μου πόρτας ήταν διεμφυλικό. άτομο.

Ε1.7 Εάν το παιδί μου μου μου έφερνε στο σπίτι έναν φίλο που είναι διεμφυλικό άτομο, θα ένιωθα άνετα να έχω το άτομο αυτό μέσα στο σπίτι μου.

Ε 1.8 Εάν κάποιος τον οποίο γνώριζα πολύ καιρό μου αποκάλυπτε ότι είναι διαφορετικού φύλου, αυτό θα με αναστάτωνε.

Ε1.9 Εάν μάθαινα ότι κάποιος είναι διεμφυλικό άτομο, θα προσπαθούσα να τον αποφύγω.

Ε1.10 Εάν ένα διεμφυλικό άτομο μου ζητούσε να γίνει συγκάτοικός μου, θα ήθελα να αρνηθώ.

Ε1.11 Δεν θα αισθανόμουν άνετα εάν αντιλαμβανόμουν ότι είμαι μόνος μαζί με ένα διεμφυλικό άτομο.

Ε1.12 Θα αισθανόμουν άνετα εάν εργαζόμουν σε μια εταιρεία όπου τα διεμφυλικά άτομα είναι ευπρόσδεκτα.

Ε1.13 Εάν κάποιος τον οποίο γνώριζα μου αποκάλυπτε ότι είναι διεμφυλικό άτομο, πιθανότατα δεν θα ήμουν πλέον τόσο κοντά σε αυτό το άτομο.

Ε1.14 Εάν μάθαινα ότι ο γιατρός μου είναι διεμφυλικό άτομο, θα ήθελα να ψάξω για άλλον γιατρό.

**ΠΑΡΑΓΟΝΤΑΣ 2 (Πεποιθήσεις για το βιολογικό και το κοινωνικό φύλο)**

Ε2.1 Ένα άτομο που δεν είναι σίγουρο εάν είναι άνδρας ή γυναίκα είναι ψυχικά άρρωστο.

Ε2.2 Εάν κάποιος είναι άνδρας ή γυναίκα εξαρτάται από το εάν αισθάνεται άνδρας ή γυναίκα.

Ε2.3 Εάν γεννήθηκες άνδρας, ό,τι και να κάνεις αυτό δεν αλλάζει.

Ε2.4 Εάν κάποιος είναι άνδρας ή γυναίκα εξαρτάται κυρίως από τα εξωτερικά γεννητικά του όργανα.

Ε2.5 Ο άνθρωπος ή είναι άνδρας ή γυναίκα. Δεν υπάρχει κάτι ενδιάμεσο.

Ε2.6 Εάν ένα διεμφυλικό άτομο προσδιορίζεται ως γυναίκα, θα πρέπει να έχει δικαίωμα να παντρευτεί έναν άνδρα.

Ε2.7 Αν και οι περισσότεροι άνθρωποι είναι άνδρες ή γυναίκες, υπάρχουν και άνθρωποι που είναι κάτι ενδιάμεσο.

Ε2.8 Όλοι οι ενήλικες θα πρέπει να προσδιορίζονται είτε ως άνδρες είτε ως γυναίκες.

Ε2.9 Ένα παιδί που γεννιέται με αμφίβολα γεννητικά όργανα θα πρέπει κατευθύνεται στο να γίνει είτε άνδρας είτε γυναίκα.

Ε2.10 Ένα άτομο δεν θα πρέπει οπωσδήποτε να είναι καθαρά άνδρας ή γυναίκα για να είναι φυσιολογικό και υγιές.

**ΠΑΡΑΓΟΝΤΑΣ 3 (Ανθρώπινη αξία)**

Ε3.1 Τα διεμφυλικά άτομα είναι ανθρώπινα όντα με αξία ανεξάρτητα από το πως βλέπω εγώ το να είσαι διεμφυλικό άτομο.

Ε3.2 Τα διεμφυλικά άτομα πρέπει να τα μεταχειρίζεται κανείς με τον ίδιο σεβασμό και αξιοπρέπεια όπως κάθε άλλο άτομο.

Ε3.3 Θα εύρισκα εντελώς απαράδεκτο να βλέπω να πειράζουν ή να κακομεταχειρίζονται ένα διεμφυλικό άτομο.

Ε3.4 Τα διεμφυλικά άτομα είναι ανθρώπινα όντα τα οποία δίνουν και αυτά τον δικό τους αγώνα όπως όλοι εμείς οι υπόλοιποι.

Ε3.5 Τα διεμφυλικά άτομα θα πρέπει να έχουν και αυτά την ίδια δυνατότητα να έχουν ένα σπίτι να στεγάζονται όπως κάθε άλλο άτομο.

**Οι** **απαντήσεις κατανέμονται σε κλίμακα Likert 7 σημείων η οποία εκτείνεται από το «διαφωνώ έντονα» έως το «συμφωνώ έντονα».**

**ΣΗΜΑΝΤΙΚΟ: Στις παρακάτω ερωτήσεις (items) απαντήσεις κατανέμονται σε κλίμακα Likert 7 σημείων η οποία εκτείνεται από το «συμφωνώ έντονα» έως το «διαφωνώ έντονα» (δηλαδή ανάστροφα):**

**Παράγοντας 1: E3,E4,E8,E9,E10,E11,E13,E14.**

**Παράγοντας 2: E1,E3,E4,E5,E8,E9.**
